# The effect of 4-hexylresorinol administration on NAD+ level and SIRT activity in Saos-2 cells

**DOI:** 10.1186/s40902-021-00326-2

**Published:** 2021-11-01

**Authors:** In-Song Lee, Jun-Ho Chang, Dae-Won Kim, Seong-Gon Kim, Tae-Woo Kim

**Affiliations:** 1 Department of Orthodontics, School of Dentistry, Seoul National University, Seoul, 3080 Republic of Korea; 2 Department of Oral Biochemistry, College of Dentistry, Gangneung-Wonju National University, Jibyun-dong, Gangneung, Gangwondo 28644 Republic of Korea; 3 Department of Oral and Maxillofacial Surgery, College of Dentistry, Gangneung-Wonju National University, Jibyun-dong, Gangneung, Gangwondo 28644 Republic of Korea

**Keywords:** 4-hexylresorcinol, Sirtuin, NAD+

## Abstract

**Background:**

4-hexylresorcinol (4HR) has been shown to have anti-oxidant activity similar to that of resveratrol. As resveratrol increases sirtuin (SIRT) activity, 4HR might behave similarly to resveratrol.

**Method:**

In this study, the expression levels of SIRT1, SIRT3, and SIRT6 were evaluated after 4HR administration (1–100 μM). As NAD+ is a substrate for SIRTs, its levels with SIRT activity were also studied.

**Results:**

In the results, SIRT3 (100 μM at 24 h) and SIRT6 (1–100 μM at 24 h and 10 μM at 8 h) were shown to have significantly higher expression levels compared to untreated control (*p* < 0.05). Pan-SIRT activity and the NAD+ level was significantly increased compared to that of the untreated control (*p* < 0.05; 10 and 100 μM at 24 h).

**Conclusion:**

4HR administration increased SIRT activity and the NAD+ level in Saos-2 cells.

## Background

Most clinicians expect successful treatment outcomes after their practice. However, various dental procedures such as oral surgery and orthodontic tooth movement can apply stress to the teeth and surrounding dental tissue including alveolar bone. If tissue fails to manage the stress from surgical treatment, wound healing will be delayed and result in inflammation [1, 2]. Although it is a rare incident, it also has been reported that pulpal side effects and root resorption can occur secondary to the orthodontic tooth movement [3]. Excessive stress is known to increases the production of reactive oxygen species (ROS) which is a main reason of inflammation [4–6]. Therefore, the application of anti-oxidant may be beneficial preventing the complications from surgical [5] or orthodontic treatment [3].

Resveratrol is a natural polyphenol and has anti-oxidant activity [7]. Similar to resveratrol, 4-hexylresorcinol (4HR) is a phenolic compound, but synthetic [8]. However, other resorcinolic lipids can be found in micro-organisms and plants [8]. The anti-oxidant effect of 4HR has been confirmed in lymphocytes [4], macrophages [5], and dental pulp cells [6]. When compared to resveratrol, the anti-oxidant activity of 4HR has a similar performance [4–6].

Anti-oxidant activity is associated with scavenging ROS from cells [4]. Sirtuins (SIRTs) are class III histone deacetylase, and their activity is related to relieving cellular stress and extending the lifespan [9]. Mammals have seven isoforms of SIRT (SIRT1 to 7). SIRTs are mainly found in the nucleus and mitochondria [9]. Specifically, SIRT3 is usually found in the mitochondrial compartment, but SIRT 1 and SIRT6 are in the nucleus [10]. Though there have been some conflicting results, resveratrol increases SIRT expression and activity [11]. 4HR has a chemical chaperone feature [8, 12]. The administration of 4HR on micro-organisms induces a dormancy-like status [12]. Micro-organisms undergoing a dormancy state are much more resistant to the outer environment and can extend their lifespan [12]. Based on these observations, 4HR administration might increase SIRT activity.

The objective of this study was to evaluate SIRTs’ expression and their activity in Saos-2 cells. First, the expression levels of SIRT1, 3, and 6 were evaluated after 4HR ad-ministration (1–100 μM). Second, pan-SIRT activity and SIRT1 activity were evaluated. As nicotinamide adenine dinucleotide+ (NAD+) is a substrate for SIRTs, its level and NAD+/NADH ratio were evaluated.

## Methods

### Cell cultures

Saos-2 cells were purchased from Korean cell line bank (Seoul, Republic of Korea). Cells were grown in 6-wells culture plates in a humidified CO_2_ incubator at 37 °C. The medium was Roswell Park Memorial Institute 1640 (RPMI 1640) medium (ThermoFisher Scientific, Waltham, MA, USA) supplemented with 10% fetal bovine serum (FBS) and antibiotics (1% penicillin–streptomycin).

### Western blot

4HR was solubilized in 0.1% dimethyl sulfoxide. When Saos-2 cells were grown to approximately 70% confluence, the cells were treated with 1, 10, and 100 μM 4HR for 2, 8, or 24 h; control cells were treated with 0.1% dimethyl sulfoxide in culture medium. Cultured cells were harvested with 0.01% trypsin and 1 mM ethylene-diamine-tetra-acetic acid. Cellular lysis was carried out with protein lysis buffer (PRO-PREPTM, iNtRON Biotechnology INC, Sungnam, Republic of Korea). Collected lysates underwent Western blotting for SIRT1, SIRT3, and SIRT6. Antibodies against SIRTs were purchased from Abcam (Cambridge, UK). The quantification of the proteins was performed as described previously [5].

### Pan-SIRT activity assay and NAD+ assay

Pan-SIRT activity was measured using a universal SIRT activity assay kit (CAT: ab156915, Abcam). Cells received 1, 10, and 100 μM 4HR, and cellular lysates were collected after 2, 8, and 24 h. The subsequent procedure was in accordance with the manufacturer’s protocol. In brief, 50 μL of diluted capture antibody was added to each well, and the plate was incubated for 60 min. After washing 3 times, 50 μL of diluted detection antibody was applied to each well, and the plate was incubated for 30 min. Then, the well was washed 4 times. For signal detection, 100 μL of developer solution was applied, and the plate was incubated for 5 min. To stop the reaction, 100 μL of stop solution was applied, and the absorbance was measured at 450 nm. The SIRT activity (OD/min/mg) was calculated using the following formula. The protein amount is expressed in micrograms, and min signifies incubation time in minutes.

$$ \mathrm{Universal}\kern0.5em \mathrm{SIRT}\kern0.5em \mathrm{Activity}=\left(\frac{\mathrm{Sample}\kern0.5em \mathrm{OD}-\mathrm{NNC}\kern0.5em \mathrm{OD}}{\Pr \mathrm{oteinAmount}\times \min}\right)\times 1000 $$

The NAD+ assay was performed with an NAD+/NADH assay kit (CAT: ab65348, Abcam). Cells received 1, 10, and 100 μM 4HR, and cellular lysates were collected after 2, 8, and 24 h. For NADH measurements, the samples were heated to 60 °C for 30 min to decompose NAD. For the measurement of total NAD+/NADH, the heating step was omitted. Then, the samples were cooled on ice. The reaction mix was added to the samples and incubated for 5 min at room temperature to convert NAD+ to NADH. NADH developer was added, and the plate was incubated for 1 h during reaction cycles. Using a microplate reader, the absorbance was measured multiple times during the 1–4 h incubation.

### Statistical analysis

Numeric data were shown as average ± standard deviation. Independent samples *t* tests were used for the comparison of two independent groups. For the comparison of more than three independent groups, analysis of variance was used. For post hoc tests, Bonferroni’s method was used. The statistically significant level was set as *p* < 0.05.

## Results

### 4HR increased SIRT3 and SIRT6 expression

**Fig. 1:**
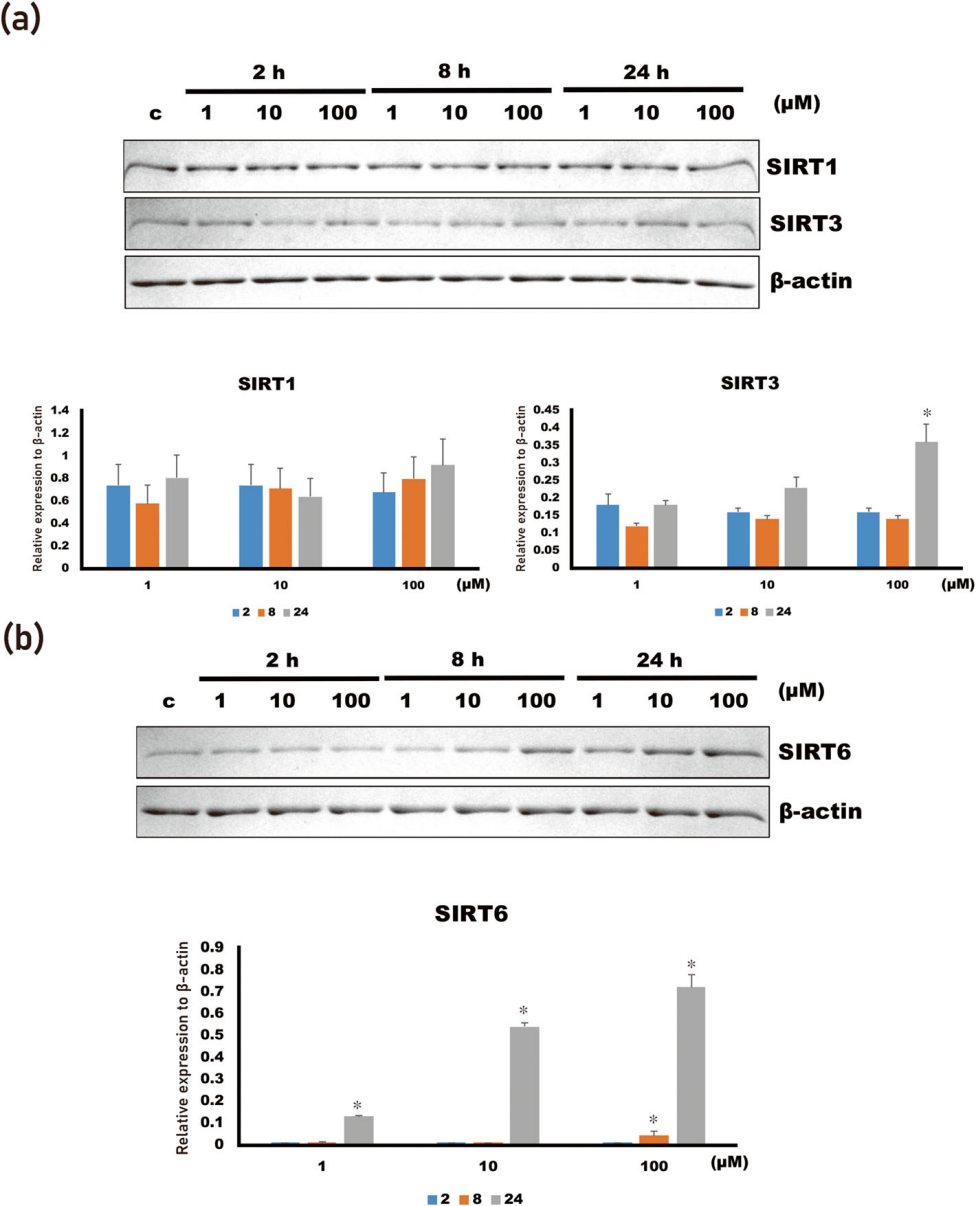
Western blot for SIRT1, 3, and 6. **a** The expression level of SIRT1 was not significantly changed by 4HR administration (1*–*100 μM). However, the expression level of SIRT3 was significantly increased in the 100 μM 4HR group at 24 h after administration (*p < 0.05). **b** The expression level of SIRT6 was significantly increased in the 1*–*100 μM 4HR group at 24 h after administration and in the 100 μM 4HR group at 8 h after administration (*p < 0.05)

The difference in the relative expression level of SIRT1 among the groups was not statistically significant (Fig. 1a; *p* > 0.05). However, a difference in the relative expression levels of SIRT3 and SIRT6 among the groups was statistically significant (Fig. 1a, b; *p* < 0.001). In the post hoc test, the expression level of SIRT3 was significantly increased in the 100 μM 4HR group compared to untreated control at 24 h after administration (*p* < 0.001). The expression level of SIRT6 was significantly increased in the 1, 10, and 100 μM 4HR groups compared to that of the untreated control at 24 h after administration (*p* = 0.001 for 1 μM 4HR group and < 0.001 for 10 and 100 μM 4HR groups). In addition, it was significantly increased in the 100 μM 4HR groups compared to the untreated control at 8 h after administration (*p* < 0.001).

### 4HR increased pan-SIRT activity and NAD+ level

**Fig. 2:**
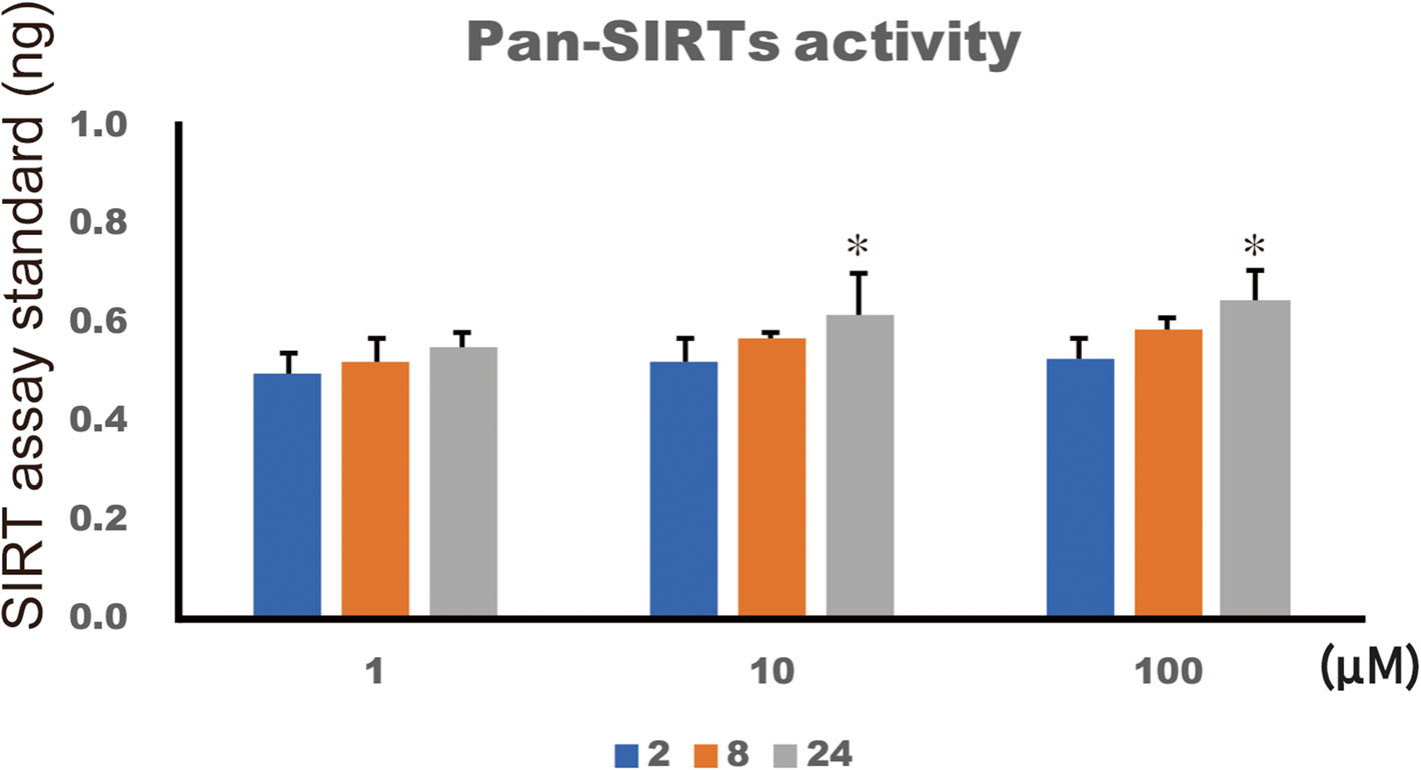
Pan-SIRT activity. Pan-SIRT activity was significantly increased in the 10 and 100 μM 4HR groups at 24 h after administration compared to that of the untreated control (*p < 0.05)

The difference in pan-SIRT activity among the groups was statistically significant (Fig. 2; *p* < 0.001). In the post hoc test, pan-SIRT activity in the 10 and 100 μM 4HR groups was significantly higher compared to that of the untreated control at 24 h after administration (*p* = 0.010 and 0.001, respectively).

**Fig. 3:**
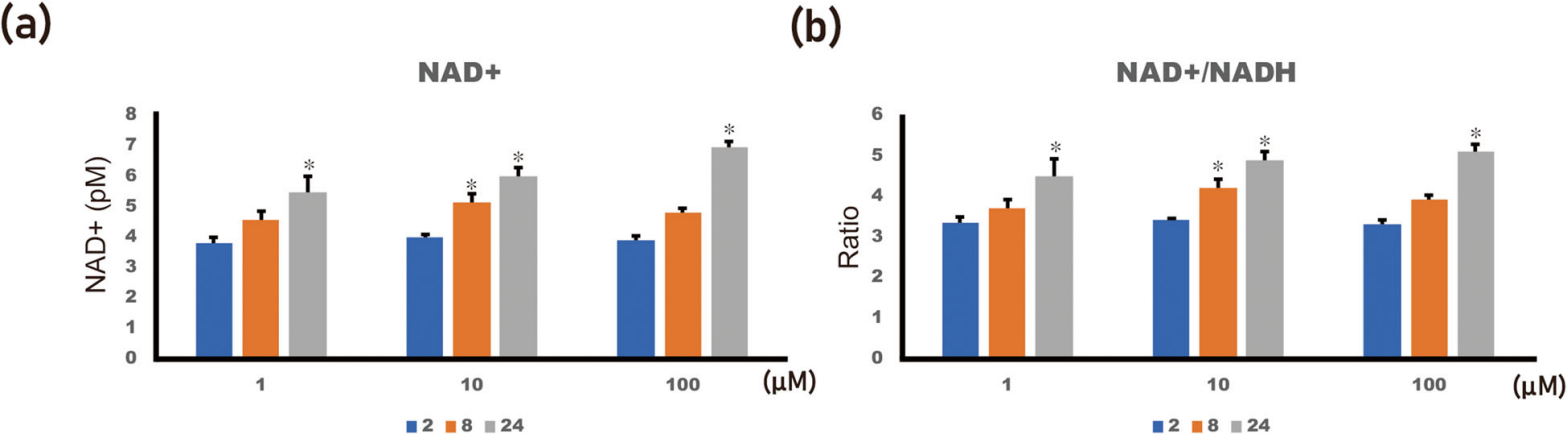
NAD+ level. **a** The NAD+ level was significantly increased in the 1, 10, and 100 μM 4HR groups at 24 h after administration compared to that of the untreated control (*p < 0.05). Additionally, it was significantly increased in the 10 μM 4HR groups at 8 h after administration compared to untreated control (*p < 0.05). **b** The NAD+/NADH ratio was significantly increased in the 1, 10, and 100 μM 4HR groups at 24 h after administration compared to that of the untreated control (*p < 0.05). Additionally, it was significantly increased in the 10 μM 4HR groups at 8 h after administration compared to untreated control (*p < 0.05)

The difference in NAD+ levels among groups was statistically significant (Fig. 3a; *p* < 0.001). In the post hoc test, the NAD+ level in the 1, 10, and 100 μM 4HR groups was significantly higher compared to that of the untreated control at 24 h after administration (*p* < 0.001). Additionally, the NAD+ level in the 10 μM 4HR groups was significantly higher compared to that of the untreated control at 8 h after administration (*p* = 0.003). The difference in NAD+/NADH ratio among the groups was statistically significant (Fig. 3b; *p* < 0.001). In the post hoc test, the NAD+ level in the 1, 10, and 100 μM 4HR groups was significantly higher compared to that of the untreated control at 24 h after administration (*p* = 0.001 for 1 μM 4HR group and < 0.001 for 10 and 100 μM 4HR groups). Additionally, the NAD+ level in the 10 μM 4HR groups was significantly higher compared to that of the untreated control at 8 h after administration (*p* = 0.034).

## Discussion

Sirtuins are NAD+-dependent enzymes and are important for resistance to cellular stress. In this study, the expression of SIRT3 and 6 in Saos-2 cells was increased by 4HR administration (1–100 μM) (Fig. 1). The administration of 4HR increased pan-SIRT activity (Fig. 2). Additionally, the NAD+ level and NAD+ to NADH ratio were also in-creased by 4HR administration (Fig. 3). In conclusion, the administration of 4HR on Saos-2 cells showed increased SIRT3 and 6 expressions and pan-SIRT activity via an in-creased NAD+ to NADH ratio.

Prototype SIRTs are found in yeast, and their expression is associated with extension of the lifespan [13]. When the cellular metabolism is restricted, the expression level of SIRT1 is increased in the muscles [14]. SIRT3 is mainly expressed in mitochondria and associated with energy metabolism [15]. SIRT6 inhibits glucose uptake and its metabolism [16]. In this study, the administration of 4HR increased SIRT3 and SIRT6 in Saos-2 cells (Fig. 1). According to our recent study, 4HR administration reduced mitochondrial respiratory activity and ATP synthesis in human umbilical vein endothelial cells (HUVECs) [17]. Therefore, 4HR-mediated inhibition of mitochondrial activity might be associated with increased SIRT3 and SIRT6 expression.

Reduced mitochondrial activity by 4HR administration might be fatal to cancer cells. Indeed, 4HR administration has been shown to have anti-cancer activity [18, 19]. Though the administration of 4HR would inhibit the growth of tumor, elevated SIRT3 and SIRT6 could play a protective role for cancer cells that survived after 4HR treatment, and these cancer cells might be more resistant to other cytotoxic cancer therapy. Superoxide dismutase (SOD) is activated by 4HR administration in RAW264.7 cells [5]. SOD is also activated by SIRT3 activation [20].

Interestingly, 4HR administration increased the SIRT3 expression level (Fig. 1a). SIRT6 reduces pancreatic inflammation and fatty liver formation induced by a high-fat diet [21]. SIRT6 decreases aging-associated inflammatory reactions via inhibiting the nuclear factor kappa B (NF-κB) pathway [22]. 4HR administration increased the SIRT6 expression level in this study (Fig. 1b), and 4HR is strong inhibitor of the NF-κB path-way [23, 24]. The administration of 4HR decreases the expression of NF-κB, while increases ikappaB kinase in HUVECs [25].

The level of NAD+ is associated with cellular stress. When the available nutrients are restricted, AMP-activated protein kinase (AMPK) is activated, and the NAD+ level is in-creased as a consequence [26]. Fibroblast growth factor 21 administration activates SIRT1 and reduces body weight [27]. The activity of SIRTs can be regulated by the NAD+ level [28]. In addition to SIRTs, poly(ADP-ribose) polymerases (PARPs) and cyclic ADP-ribose synthases also use NAD+ as an enzyme substrate [9, 11]. As PARPs are DNA repair enzymes and highly expressed in cancer, their inhibitor can be used in cancer treatment [29]. PARPs are the main consumers of NAD+, and their inhibition may result in SIRT activation via flooding NAD+. However, the relation between PARPs and 4HR administration is yet to be clarified. In this study, 4HR administration increased the NAD+ level and NAD+/NADH ratio (Fig. 3).

4HR has been developed as an antiseptic [30]. As the derivatives of resorcinolic lipid suppress microbial proliferation, the application of 4HR can inhibit microbial growth [8]. When 4HR is prescribed for oral intake, its absorption rate from the gastro-intestinal tract is poor [31]. When people receive 4HR per os, only 18% of the initial dosage is found in the urine within the first 12 h [31]. Due to the poor absorption rate, it has been pre-scribed for killing intestinal pathogens [32]. Russian scientists found that micro-organism surviving 4HR administration undergo dormancy and are more resistant to outer environmental stress [12]. Therefore, 4HR has been considered as a chemical chaperone. A 4HR-mediated increase in SIRT activity and NAD+ level might be associated with its chaperone-like activity.

The possible anti-oxidant activity of 4HR might suggest that its applications can be useful in various conditions from orthodontic and surgical treatments. These conditions include gingivitis [33], root resorption [3], and dental pulp necrosis [6] during orthodontic treatment and several studies have reported that anti-oxidant activity can affect the expression of their manifestation [4, 5]. The application of 4HR ointment accelerates diabetic burn wound healing via improved capillary regeneration [34]. Especially, the fact that 4HR has been proven to be safe as food additive for a long period [31] may make its utilization more promising.

A limitation of current study was that there are seven analogues of mammalian SIRTs. However, only SIRT1, 3, and 6 expression levels were evaluated after 4HR administration. The expression levels of SIRT2, 4, 5, and 7 should also be evaluated. According to our recent study on HUVECs, SIRT2, 4, 5, and 7 mRNA levels were increased by 4HR administration in HUVECs [17]. In this study, pan-SIRT activity was increased by 4HR administration (Fig. 2). As individual SIRTs’ activity might be different after 4HR administration, individual enzyme activity after 4HR administration should also be studied in following projects. In this study, Saos-2 cell was used for evaluation. Saos-2 cell is originated from osteosarcoma cell and has been used for human osteoblastic cell [35]. As it was cancer cell, additional cellular experiment using primary cultured human cell would be required for the confirmation of current finding.

## Conclusion

In this study, the administration of 4HR on Saos-2 cells increased the NAD+ level and NAD+/NADH ratio. The expression of SIRT3 and SIRT6 was also increased by 4HR administration.

## Data Availability

Data sharing is not applicable to this article since no dataset was generated or analyzed during the current study.

## Abbreviations

4HR: 4-hexylresorcinol
ROS: Reactive oxygen species
SIRT: Sirtuin
NF-κB: Nuclear factor-kappa B
HUVECs: Human umbilical vein endothelial cells
NAD: Nicotinamide adenine dinucleotide
